# Case report of incidental and asymptomatic appendiceal cavernous hemangioma

**DOI:** 10.1016/j.ijscr.2025.111492

**Published:** 2025-06-09

**Authors:** Mislav Rakić, Mario Soldo, Ante Šabić, Iva Škifić

**Affiliations:** a Department of Abdominal Surgery, Clinical Hospital Dubrava, Zagreb, Croatia; b Department of Pathology, Clinical Hospital Dubrava, Zagreb, Croatia

**Keywords:** Appendiceal hemangioma, Incidental, Asymptomatic, Literature review

## Abstract

**Introduction:**

Appendix hemangiomas are extremely rare – only a few reported cases exist in published literature.

**Case presentation:**

Here we present the case of incidental appendiceal hemangioma that was discovered upon surgery for unrelated colon cancer. An atypical right hemicolectomy was performed as treatment of this unsuspected pathology.

**Discussion:**

Other than in the liver, cavernous hemangiomas are rare intra or retroperitoneal tumors. Only three cases of appendiceal hemangioma exist in published literature and this is the first one that was asymptomatic. Accordingly, there are no tools for making preoperative diagnosis. Focal calcifications and a thickened appendix on computed tomography scans may indicate hemangioma even in this unusual location.

**Conclusion:**

We believe that appendectomy or atypical ileocecal resection of the colon is the method of choice for treatment of appendiceal hemangioma due to its non-metastatic potential.

## Highlights

• Appendiceal hemangiomas are extremely rare.
• This is a first reported case of asymptomatic appendiceal hemangioma.
• Appendectomy if possible, or ileocecal resection is proposed method of surgical care.

This work was written in accordance with the SCARE guidelines [1].

## 1 Introduction

Hemangiomas are benign vascular tumors that are commonly found in various organs such as liver, colon, small bowel, pancreas and extraabdominaly [[2], [3], [4], [5], [6], [7], [8], [9], [10]]; however, their occurrence in the appendix is exceedingly rare. This report presents a case involving a male patient who underwent surgery for colon cancer, during which an appendiceal hemangioma was unexpectedly identified. In this report, we aim to present our method of surgical treatment for the discovered appendiceal hemangioma, emphasizing the surgical approach taken and the outcomes achieved.

## 2 Case presentation

A 77 year-old patient was referred from a general hospital for surgical intervention due to a diagnosis of left colic flexure carcinoma. Colonoscopy revealed a lumen-occluding lesion located 85 cm from the anocutaneous border, and biopsy results confirmed the presence of adenocarcinoma. Multislice computer tomography (MSCT) of the thorax, abdomen, and pelvis demonstrated segmental thickening of corresponding colon segment accompanied by locoregional lymphadenopathy; notably, there were no signs of metastatic disease. Preoperatively, laboratory values were largely within normal limits aside from moderate normocytic anemia (hemoglobin 93 g/L, MCV 90 fL). Tumor markers were found to be in the normal range (CEA 3.7, CA19-9<2).

The patient's medical history is significant for type II diabetes, hypertension, gastroesophageal reflux disease (GERD), and benign prostate hyperplasia (BPH). He had previously undergone two surgical procedures for biologic aortic valve replacement.

**Fig. 1:**
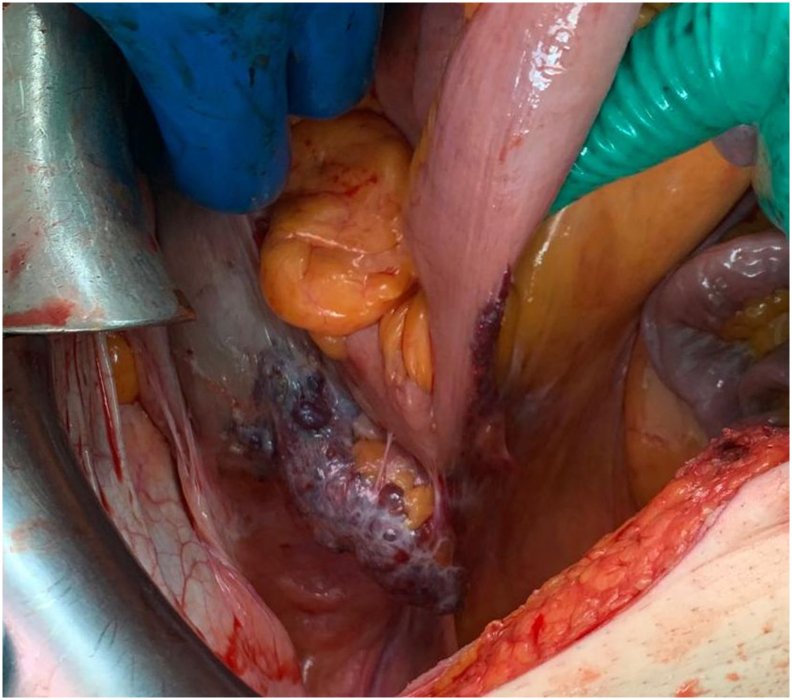
Intraoperative findings.

The surgical approach employed was an open midline laparotomy. Upon exploration, a large tumor mass consistent with adenocarcinoma was identified. However, examination of the appendix revealed a purple, tense, grape-like mass extensively involving the appendix and extending into the terminal ileum for several centimeters (Fig. 1). A standard left hemicolectomy with latero-lateral colocolic anastomosis was performed to address adenocarcinoma. Regarding the appendiceal tumor, an atypical right hemicolectomy was conducted, incorporating the terminal division of the ileocolic artery due to the inclusion of at least the first level of mesocolonic lymph nodes. No palpable lymphadenopathy was detected at the ileocolic mesenteric root which was preserved. Reconstruction of the alimentary tract was achieved via a latero-lateral ileocolic anastomosis.

**Fig. 2:**
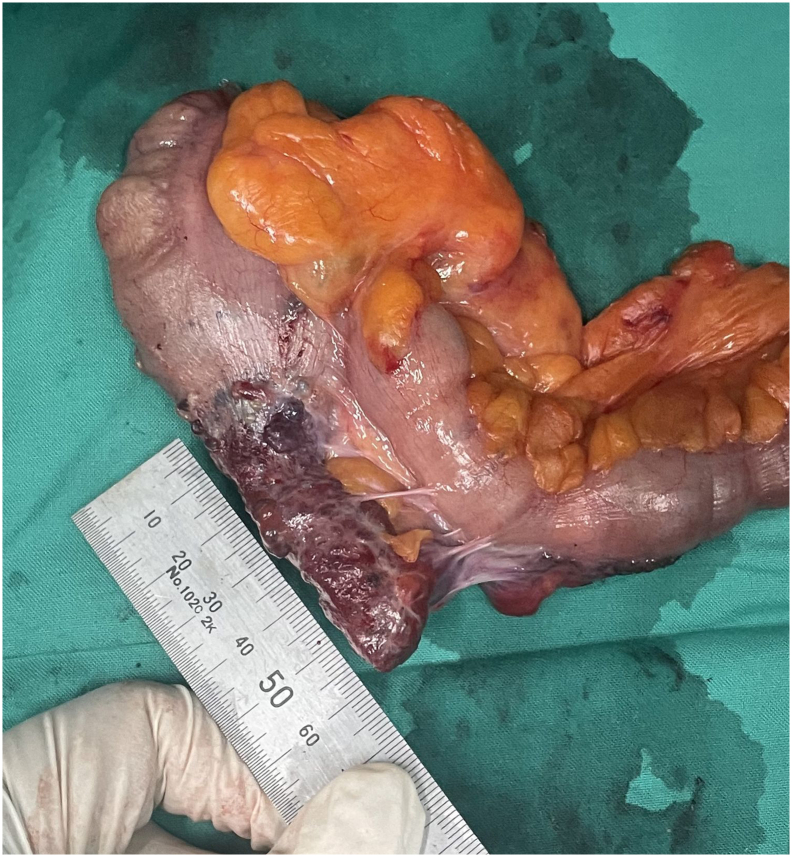
Specimen for histologic evaluation.

**Fig. 3:**
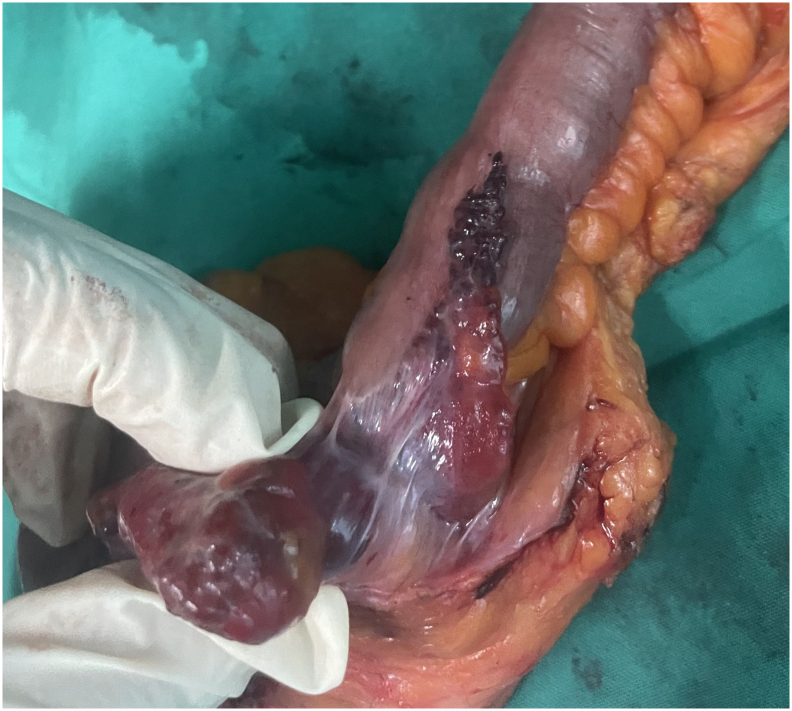
Lateral view with ileal extension.

**Fig. 4:**
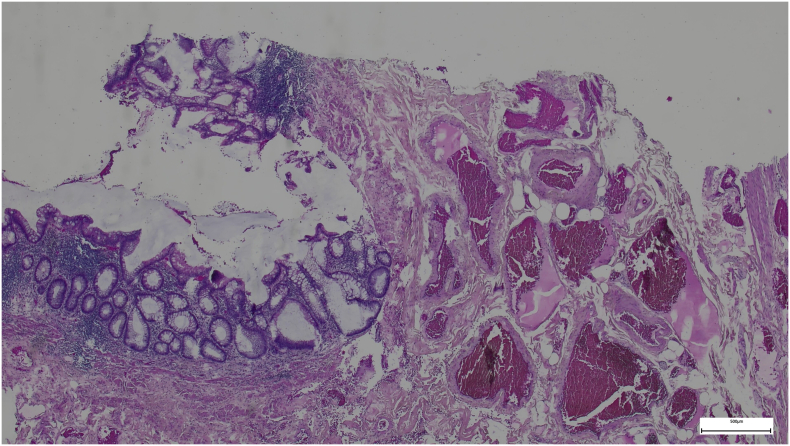
Histology specimen, hematoxylin and eosin stain.

**Fig. 5:**
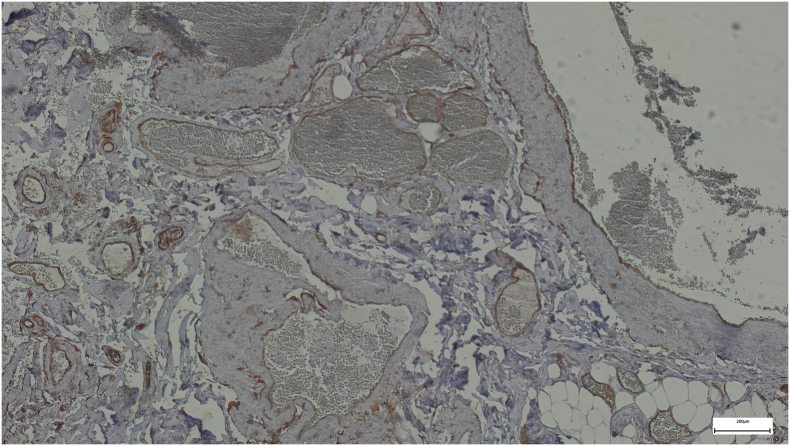
CD34 immunohistology.

Surgical specimens were obtained for patohistologic examination (Fig. 2, Fig. 3). The left colic specimen was characterized as pT3N1b adenocarcinoma exhibiting perineural invasion (PNI), lymphovascular invasion (LVI), and two (of 20 harvested) lymph node metastases. The right colic specimen consisted of 20 cm of ileum in continuity with 5 cm of cecum and a 5 cm long appendix, 1.5 cm wide, which was noted to have been extensively infiltrated by tumor tissue, exhibiting nodular, strawberry-like lesions that extended to the orifice. Histopathological assessment revealed normal mucosa, devoid of atypical epithelial cells, whereas the submucosal, muscular, and subserosal layers were markedly replaced by dilated vascular spaces lined with immunoreactive CD34-positive cells (Fig. 4, Fig. 5). Eight lymph nodes were excised, demonstrating reactive changes without evidence of tumor proliferation. Consequently, the histological findings were consistent with a diffuse cavernous hemangioma.

The patient experienced an uneventful recovery and was discharged on the sixth postoperative day.

## 3 Discussion

Other than in the liver, hemangiomas of abdominal organs and retroperitoneum are very rare tumors.

Hepatic hemangiomas are the most common benign tumors of the liver, with an estimated incidence ranging from 1 % to 20 % of the general population, often discovered incidentally during imaging for other reasons. Diagnosis typically involves imaging techniques such as ultrasound, CT scans, or MRI, which can help differentiate them from malignant lesions, and treatment is generally not required for asymptomatic hemangiomas, while symptomatic cases may necessitate surgical intervention; alternative therapies such as liver transplantation, radiofrequency ablation, transarterial embolization, and transarterial chemoembolization have also been gaining in importance. Follow-up usually involves periodic imaging to monitor for any changes in size or symptoms, although most hemangiomas remain stable and do not require aggressive management [2].

In the large bowel, they are most commonly found in the rectosigmoid segment and can be associated with Osler-Weber-Rendu, Maffucci's, and Klippel-Trenaunay syndromes [3]. Hemangiomas in these locations are prone to bleeding, which is the most common cause of hospitalization. Medical options targeting VEGF (vascular endothelial growth factor) and FGF (fibroblast growth factor) mediated pathways, such as bevacizumab and thalidomide, are therapeutic options, as are endoscopic approaches like sclerotherapy and electrocautery; only curative treatment is surgical excision [[4], [5], [6]].

Small bowel hemangioma is a rare disease. Usually, it clinically presents as occult gastrointestinal bleeding (OGIB). The etiology of OGIB is dominated by vascular abnormalities (angiodysplasia, telangiectasia), which account for 70–80 % of the cases, followed by small intestine tumors that account for up to 10 % [7]. The rarity of these tumors is clearly evident in a paper by Iordache et al. [8]–at the time of writing, there were 22 articles in the literature, all case reports. In the literature review, most patients presented with acute or chronic anemia and had melena or hematochesia. All but 3 were surgically treated with resection.

Retroperitoneal hemangiomas are extremely rare in adulthood. In a paper by Hou et al. [9] there are 38 hemangiomas described in the literature review, 6 of these originated from adrenals, 12 from the pancreas, only 3 were indubitably primary retroperitoneal, 7 were from an unspecified site, and 1 originated from the psoas major muscle. The paper highlights the difficulties in correct diagnosis due to uncharacteristic radiologic findings. It is also emphasized that there is no need for further diagnosis and follow-up of asymptomatic cases, whereas further surgical treatment is needed when the tumor grows rapidly, oppresses adjacent organs, or develops non-specific symptoms.

Pancreatic hemangioma is a very rare type of vascular tumor. According to Jin et al. [10], only around 20 cases are reported. Operated patients had mass-related symptoms. It is proposed that parenchyma-sparing pancreatectomy is a preferred procedure due to the benign nature of the disease and local resection for selected peripherally located and well-defined lesions.

Cavernous malformations of the brain and spinal cord are a subtype of abnormal vascular configurations affecting the central nervous system (CNS); other types are arteriovenous malformations, developmental venous anomalies, and capillary telangiectasias. They may occur sporadically or in a familiar pattern. If symptomatic, they present according to location, but a substantial proportion is asymptomatic and discovered incidentally on imaging. Diagnosis of CNS cavernous malformations is based upon the characteristic radiologic appearance on MRI. Asymptomatic lesions are observed, irrespective of location, while symptomatic cavernous malformations of CNS are treated either surgically or medically [11,12].

As previously noted, there are only a handful of cases of appendiceal hemangioma documented in published literature. Harned et al. [13] reported a case of concomitant rectal and appendiceal bleeding hemangiomas in 1974, while Geramizadeh et al. [14] described a case of cavernous hemangioma that mimicked acute appendicitis in 2016. Additionally, Takagi et al. [15] reported a case of cavernous hemangioma presenting with chronic right lower quadrant pain in 2017.

**Fig. 6:**
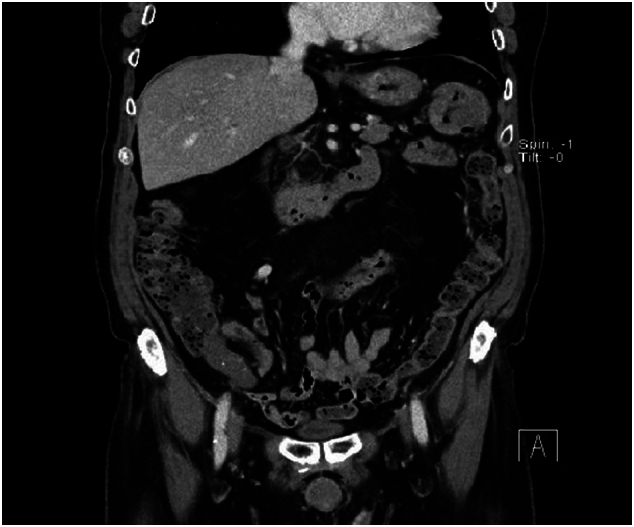
Preoperative MSCT imaging.

Due to the rarity of this pathology, there are currently no established tools for straightforward diagnosis. Upon reviewing the abdominal and pelvic CT scans of the patient in our case (Fig. 6), we observed similar characteristics previously reported by Takagi et al. [15], particularly focal calcifications of a thickened appendix, which are easily overlooked unless specially noted during the examination.

In our case, we encountered a notable tumor proliferation that exhibited benign macroscopic characteristics. Considering the laparotomy conducted for an unrelated condition and the identified localized invasion of the ileum, it was appropriate to proceed with the surgical intervention. We contend that, in the absence of invasion beyond the appendiceal borders, an appendectomy alone would have sufficed.

Currently, there is a paucity of literature addressing asymptomatic hemangiomas of the appendix. Given the challenges associated with accurate diagnosis and the rarity of the disease, a prudent approach would likely involve surgical treatment, preferentially laparoscopic, minimally comprising an appendectomy, to effectively address the tumor and exclude another, potentially malignant pathology.

## 4 Conclusion

Cavernous hemangioma of appendix is a rare disease; this is the first case of truly asymptomatic appendix-consuming hemangioma. Due to the non-metastatic potential of the tumor itself, appendectomy or ileocecal resection is the method of choice for treatment.

## Author contribution

MR operated the patient and supervised the writing of the manuscript. MS described and designed the article (corresponding author). IŠ provided pathology report and histology photograps. AŠ collected the data and discussed the content of the manuscript. All authors read and approved the final manuscript.

## Consent for publication

Written informed consent was obtained from the patient for publication of this case report and any accompanying images. A copy of the written consent is available for review by the Editor-in-Chief of this journal on request.

## Ethical approval

This kind of manuscript does not require ethical approval (exemption) by the Ethical Committee of clinical hospital Dubrava, as declared in statement of IRB mentioned above number 2025/0128-9. A copy of approval is available for review by the Editor of journal upon request.

## Guarantor

Mario Soldo.

## Research registration number

None.

## Funding

This research did not receive any specific grant from funding agencies in the public, commercial, or not-for-profit sectors.

## Declaration of Generative AI and AI-assisted technologies in writing process

During the preparation of this work the authors used Grammarly in order to improve readability and language. After using this tool, the authors reviewed and edited the content as needed and take full responsibility for the content of the publication.

## Conflict of interest statement

The authors declare that they have no conflicts of interest.
